# Uncertainty Quantification in Irreversible Electroporation Simulations

**DOI:** 10.3390/bioengineering4020041

**Published:** 2017-05-06

**Authors:** Nicholas Labarbera

**Affiliations:** Engineering Science & Mechanics, The Pennsylvania State University, State College, PA 16801, USA; nal5047@psu.edu

**Keywords:** irreversible electroporation, treatment planning, uncertainty quantification

## Abstract

One recent area of cancer research is irreversible electroporation (IRE). Irreversible electroporation is a minimally invasive procedure where needle electrodes are inserted into the body to ablate tumor cells with electricity. The aim of this paper is to investigate how uncertainty in tissue and tumor conductivity propagate into final ablation predictions used for treatment planning. Two dimensional simulations were performed for a circular tumor surrounded by healthy tissue, and electroporated from two monopolar electrodes. The conductivity values were treated as random variables whose distributions were taken from published literature on the average and standard deviation of liver tissue and liver tumors. Three different Monte Carlo setups were simulated each at three different voltages. Average and standard deviation data was reported for a multitude of electrical field properties experienced by the tumor. Plots showing the variability in the electrical field distribution throughout the tumor are also presented.

## 1. Introduction

One recent area of cancer research is irreversible electroporation (IRE). Irreversible electroporation is a minimally invasive procedure where needle electrodes are inserted into the body to ablate tumor cells with electricity. By applying an electrical field across a cell, the cell membrane becomes more permeable by developing nano-sized pores [1]. This process is called electroporation and comes in two varieties. The first is characterized by the permeability of the cell membrane only being temporarily changed and is referred to as reversible electroporation. Reversible electroporation was the first form of electroproation studied for modern medicine for the purpose of delivering new DNA cells [2]. Since then, reversible electroporation has been used for numerous drug uptake procedures [3,4,5,6]. At higher electric field strengths, the cell membrane permeability becomes permanently altered and causes the cell to die. When increasing the permeability of the cell membrane for drug or gene uptake, causing irreversible damage that results in cell death is considered an unintended consequence that should be minimized. When cell death occurs the process is called irreversible electroporation.

In 2005, Davalos et al. mathematically showed that irreversible electroporation could theoretically ablate significant amounts of tissue without detrimental thermal effects [7]. This began the research of IRE as a minimally invasive ablation technique. One of the key advantages of IRE is that tissue ablation is well defined while preserving important structures such as blood vessels and collagen [8]. The strong demarcation between living and ablated cells makes IRE a well focused ablation technique that has potential to be used for tumors adjacent to major blood vessels and other critical structures. The first report of using irreversible electroporation to ablate tumors in vivo was done in 2007 [9]. Since then, IRE has been applied to kidney tumors [10], prostate tumors [11,12], pancreatic tumors [13,14], and liver tumors [15].

When a doctor decides to use IRE, it is important to consider electrode positioning and what voltage to apply to completely ablate the tumor while minimizing the damage to healthy tissue. Numerical simulations offers information to allow doctors to make informed decisions on how to best perform IRE procedures on an individual basis for patients. Using computer models to make predictions of electrical fields for IRE treatment is an essential part of treatment planning.

A factor of safety can be included in treatment planning by applying a voltage higher than what is numerically predicted to be necessary to ablate the target area. The question though is how high should this factor of safety be set? If the safety factor is too low, then the risk of incomplete tumor ablation exists. If the factor of safety is too high, then more healthy tissue is ablated than necessary. Currently, there is no agreement on what the factor of safety should be for treatment planning [16].

We believe the factor-of-safety used in treatment planning should be a reflection of the level of uncertainty in the numerical results where the uncertainty in the numerical results are a result of the uncertainty in the tissue and tumor electrical properties. To the best of the author’s knowledge, no research has been done for uncertainty quantification of irreversible electroporation. One goal of this research is to begin to better understand how uncertainty in material properties propagate through the mathematical model and into the final solution. The hope of this research is that a greater understanding in the uncertainty of the final solution will provide physicians and researchers with more knowledge to make better informed decisions on what level the factor-of-safety should be set at.

Uncertainty quantification aims to quantitatively determine how likely an event is to occur. For the case of IRE simulations, the quantity of interest is the ablation shape. This is of interest for medical doctors because they want to choose electrode placements and voltages that ensure a high probability of ablating all cancerous cells while minimizing the ablation of healthy tissue. Before this can be done, efforts must be made to characterize the uncertainty in the model. For IRE, uncertainty comes in the model parameters such as conductivity of the tissue. To begin to understand uncertainty in IRE simulations, this research reports results from running Monte Carlo simulations for ablation of a liver tumor.

## 2. Methods

### 2.1. Governing Equations

The time required to charge the membrane of the cell is estimated on the order of nanoseconds while the pulse time for IRE treatments is on the order of microseconds [17]. Thus, when performing a numerical simulation, the electric field can be assumed to be direct current due to the capacitance of the tissue charging much faster than the time scale of the pulses [18,19]. The electric field for an electrostatic problem is found by solving

(1) ∇·(σ∇U)=0,

where σ is the conductivity and *U* is the electric potential. The electric field, E, is defined as the negative gradient of the potential,

(2) E=-∇U.

The magnitude of the electrical field is the quantity used to determine if a cell loses viability. For this reason, when performing IRE simulations the electrical field is often the quantity of interest.

If σ is a constant, then Equation (1) is linear. However, it has been shown that the conductivity of tissue increases during electroporation [20]. Modeling the conductivity as a function of the electric field,

(3) σ=σ(E),

results in Equation (1) becoming nonlinear, but also results in a more accurate model. Often a sigmoid curve for the conductivity is used [21,22,23]. If the conductivity is isotropic, then σ is a scalar.

### 2.2. Conductivity Formulation

To account for changes in conductivity from electroporation, a sigmoid Gompertz curve for the conductivity can be used [21,22,23,24]. A sigmoid Gompertz curve for conductivity has the form

(4) $$\sigma =\sigma_{0}+\frac{\sigma_{max}-\sigma_{0}}{1+exp[\frac{∥E∥-T}{W}]},$$

where T and W are coefficients used for curve fitting, $\sigma_{0}$ is the the base conductivity before electroporation, $\sigma_{max}$ is the maximum conductivity a tissue can achieve after electroporation, and ∥E∥ is the $l^{2}$-norm of the electrical field. The $l^{2}$-norm of the electrical field is defined to be

(5) $$∥E∥=\sqrt{E_{x}^{2}+E_{y}^{2}},$$

and the values of T and W are found by fitting the curve for conductivity to experimental data. In Equation (4), σ is only a function of the electrical field’s norm. The values used for T and W used are 950 V/cm and 200 V/cm respectively which are the same values used in [24].

It was stated in [25] that tissues increase conductivity between 3 to 6 times due to electroporation. A middle value of 4.5 was chosen to be used for these simulations. Therefore, for all simulations the maximum conductivity will be 4.5 times the base conductivity,

(6) $$\sigma_{max}=4.5\sigma_{0}$$

To account for uncertainty, $\sigma_{0}$, will be treated as a random variable. Consequently $\sigma_{max}$ will also be a random variable as it depends directly on $\sigma_{0}$. The distributions used will be a normal distribution. Monte Carlo simulations will be used with $\sigma_{0}$ for the tissue, $\sigma_{0}$ for the tumor, and $\sigma_{0}$ for both represented by a normally distributed random variable that will randomly vary between trials.

A recap of of the equations used can be found in Table 1.

### 2.3. Geometry

**Figure 1:**
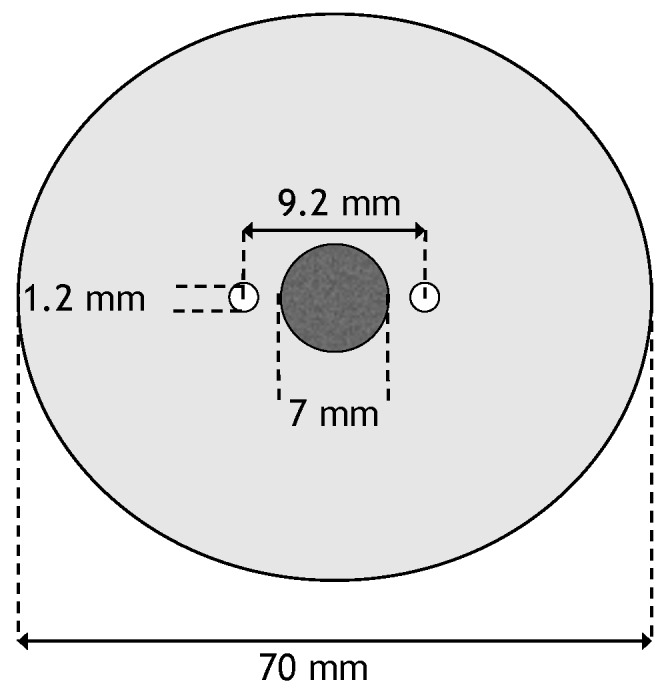
Schematic of the domain used for the simulations.

A two-dimensional geometry was used for the numerical model. It consisted of two monopolar electrodes whose centers are spaced 9.2 mm apart. In between the two electrodes is a circular tumor with diameter of 7 mm. The tumor is centered in a circular domain with a diameter of 7 cm. The electrodes have a diameter of 1.2 mm [26]. This results in a distance of 0.5 mm between the tumor and the electrodes. A schematic of the geometry can be seen in Figure 1.

### 2.4. Boundary Conditions

Boundary conditions need to be specified before Equation (1) can be solved. It is common for boundaries of the electrode to be Dirchlet type and the remaining boundaries to be Neumann type [27]. Specifically, the boundary condition for the left electrode will be

(7) $$U=V_{0},$$

where $V_{0}$ is the applied voltage of the electrode. The right electrode will be set to ground and the boundary condition would read

(8) U=0.

The other boundary of the domain will be modeled as electrically insulating by enforcing

(9) $$\frac{\partial U}{\partial n}=0,$$

where *n* is the outward pointing normal [27].

### 2.5. Monte Carlo Cases

Monte Carlo simulations were run for three different cases to evaluate how uncertainty in the conductivity propagates into predictions for the electrical field. The three different cases were:

1. Only the base conductivity of the tissue is a random variable
2. Only the base conductivity of the tumor is a random variable
3. Base conductivity of the tumor and tissue are both random variables

The Monte Carlo simulations performed here were based on the uncertainties in conductivity reported by Haemmerich et al. [28]. In their work, they reported liver tissue as having a base conductivity of 0.75 mS cm${}^{-1}$ with a standard deviation of 0.28 mS cm${}^{-1}$, and liver tumors with a base conductivity of 4.11 mS cm${}^{-1}$ with a standard deviation of 2.56 mS cm${}^{-1}$. The distributions for $\sigma_{0}$ for both tissue and the tumor will be normal distributions with mean and standard deviations as found in [28].

When using the mean values for all variables, it was found that a voltage of 1725 V is the minimum voltage necessary to completely cover the entire tumor in an electrical field strength of 700 V/cm which is a common value used as the threshold for the onset of IRE [7,24,29]. However, in treatment planning, a higher voltage than the minimum is often used as factor-of-safety. For this reason, the three cases were also run at voltages of 1800 V and 2000 V. Different voltages were also used to examine the effect voltage has on uncertainty. Each case was run with 200 trials at the three voltages. This bring the total number of different setups to 9, which resulted in 9 different data sets each consisting of 200 trials.

### 2.6. Finite Element Solver

All simulations will be performed using the commercial finite element software COMSOL. COMSOL was chosen as the simulation tool because numerous other researcher groups have chosen COMSOL to perform their IRE simulations [21,30,31,32,33,34,35]. Although other finite element softwares seem capable, COMSOL was chosen due to its ease of use and proliferation throughout the IRE research community. To handle non-linearity, Newton’s method was utilized and a direct solver was used for matrix inversion.

## 3. Results

### 3.1. Case 1: Uncertainty in Tissue Base Conductivity

First we investigate the effect of modeling the base conductivity for the healthy tissue as a random variable. The base conductivity for the liver tissue was treated as a normally distributed random variable with mean 0.75 mS cm${}^{-1}$ and standard deviation 0.28 mS cm${}^{-1}$. The base conductivity of the tumor was kept constant at 4.11 mS cm${}^{-1}$. Statistics concerning the electrical field strength experienced by the tumor were computed and are displayed in Table 2. Percentages of the tumor experiencing electrical field strengths above a given threshold are displayed in Table 3.

**Figure 2:**
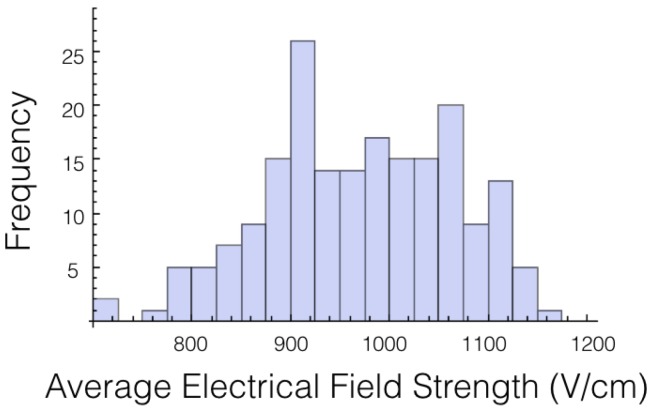
Historgam of the average electrical field experienced by the tumor at 1725 V for case 1. Roughly follows a normal distibution.

A histogram for the average electrical field experienced by the tumor for 1725 V is displayed in Figure 2. The histogram shows the shape is roughly symmetrical. Similar results were found for the other voltages and other cases.

As we are most interested in preventing tumor recurrence, we looked at the first quartile for ablation percentage. It was found that only 75% of the trials had at least 65% of the tumor experiencing an electrical field of 700 V/cm or more. For 2,000 V, all the trials but 1 completely covered the tumor in a field at least 700 V/cm. This clearly shows the need for using a safety-factor in the form of a higher voltage. The effect of using a high voltage on the amount of healthy tissue and total tissue ablated is displayed in Table 4.

**Figure 3:**
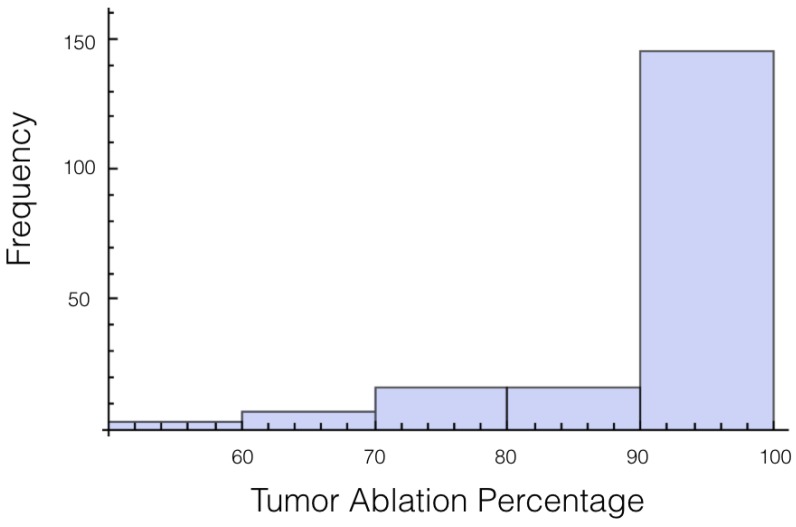
Histogram for percentage of tumor above 700 V/cm for case 1 at 1725 V.

**Figure 4:**
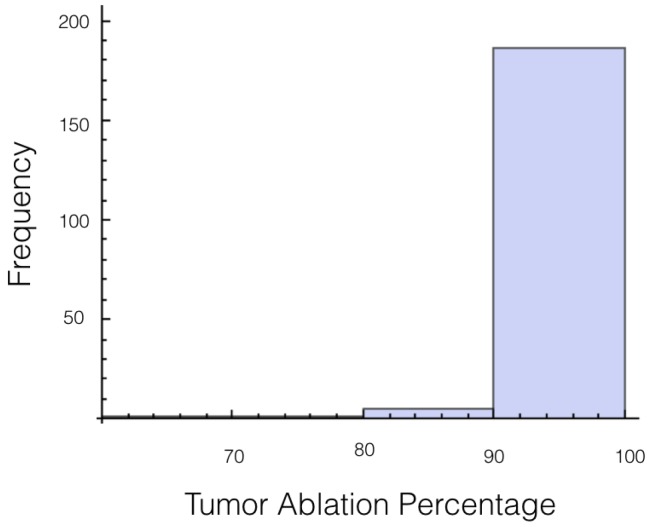
Histogram for percentage of tumor above 700 V/cm for case 1 at 2000 V.

It was also investigated to get an estimate of how many trials exposed 90% of the tumor to an electrical field strength of 700 V/cm. This was done to get an idea of the chance of recurrence by not eliminating enough of the tumor. For 1725 V, 29 out of 200 or 14.5% of the trials exposed less than 90% of the tumor to the ablating electrical field strength. This number drops to 9 out of 200 or 4.5% when a voltage of 2000 V is applied. These numbers seem high for both the 1725 V case and 2000 V case when the goal is to ensure a high enough level of tumor ablation to prevent cancer recurrence and suggest using an even higher voltage for treatment. Histograms showing ablation percentage at 700 V/cm is displayed in Figure 3 and Figure 4.

### 3.2. Case 2: Uncertainty in Tumor Base Conductivity

Next, the effect the tumor’s base conductivity had on the electrical field was investigated. This was done by modeling the conductivity in the tumor as a normally distributed random variable with mean 4.11 mS cm${}^{-1}$ and standard deviation 2.56 mS cm${}^{-1}$. The base conductivity of the tissue was kept constant at 0.75 mS cm${}^{-1}$. Results from the Monte Carlo simulations are displayed in Table 5, Table 6 and Table 7.

Interestingly, it was found that there was a lower standard deviation in the percentage of tumor above a given electrical field strength when the tumor was a random variable instead of the tissue. This result comes as a surprise since the variability of the conductivity is higher for the tumor than the tissue.

**Figure 5:**
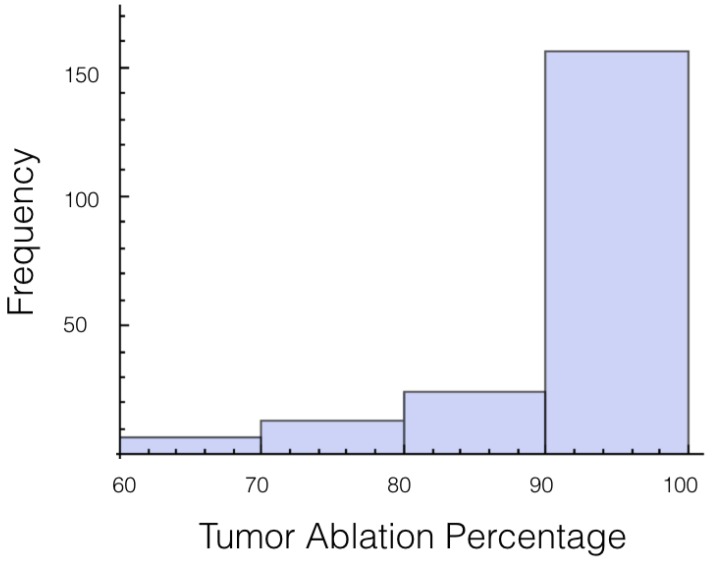
Histogram for percentage of tumor above 700 V/cm for case 2 at 1725 V.

**Figure 6:**
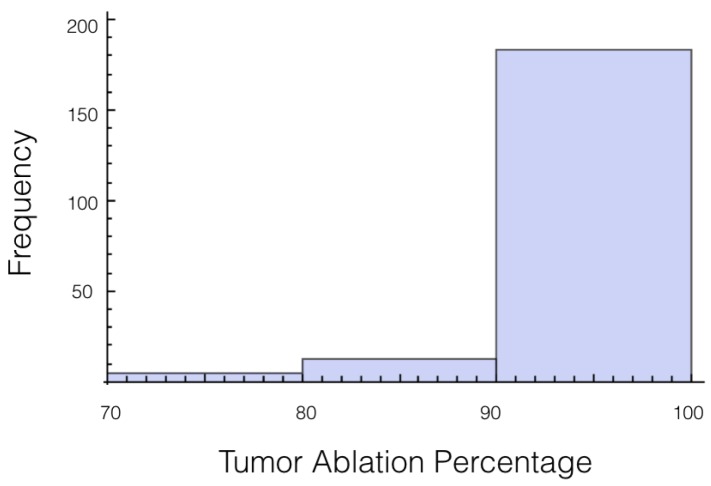
Histogram for percentage of tumor above 700 V/cm for case 2 at 2000 V.

Again, the number of trials in which 10% or more of the tumor experienced an electrical field less than 700 V/cm was calculated. At 1725 V, it was 55 out of 200 or 27.7%, and for 2000 V it was 27 out of 200 or 13.5%. The histogram for the percentage of tumor above 700 V/cm can be found in Figure 5 for 1725 V and in Figure 6 for 2000 V.

### 3.3. Case 3: Uncertainty in Tissue and Tumor Base Conductivity

**Figure 7:**
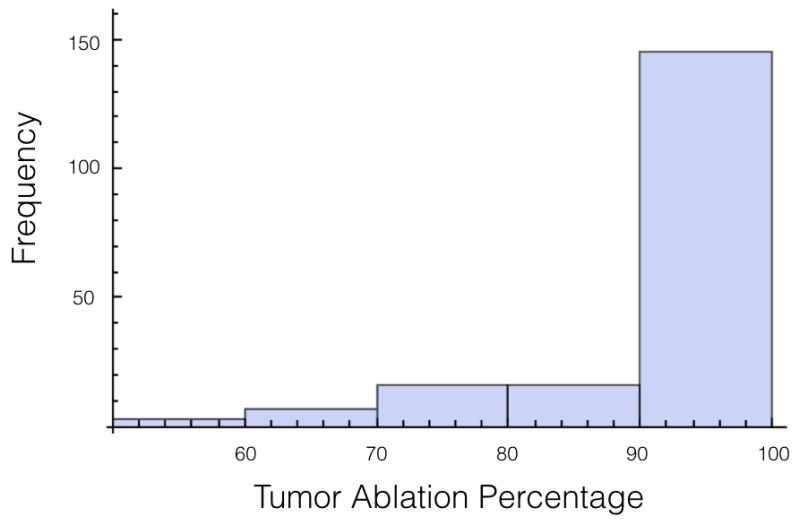
Histogram for percentage of tumor above 700 V/cm for case 3 at 1725 V.

**Figure 8:**
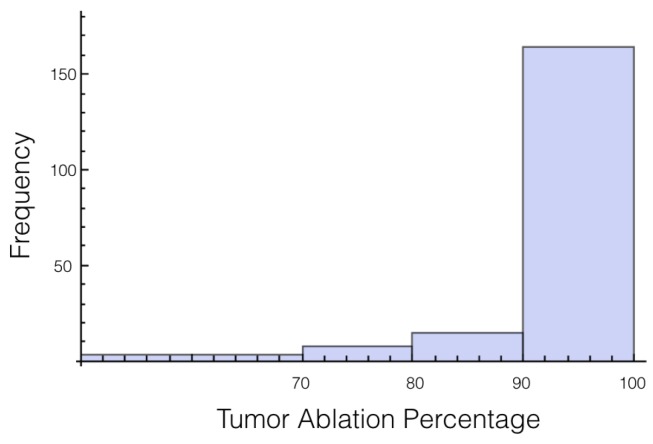
Histogram for percentage of tumor above 700 V/cm for case 3 at 2000 V.

This case best represents the scenario of treatment planning. When planning for a specific patients there is a degree of uncertainty in both the conductivity of the tissue and the conductivity of the tumor. The conductivity for the tissue and for the tumor are both treated as independent random variables whose distributions are the same as was used in cases 1 and 2. Statistics on the electrical field experience by the tumor are displayed in Table 8, Table 9 and Table 10. Also histograms for the percentage of tumor above 700 V/cm can be found in Figure 7 and Figure 8 for 1725 V and 2000 V respectively.

For a voltage of 1725 V, it was found that 10% or more of the tumor was below an electrical field strength of 700 V/cm for 55 out of the 200 trials. This number drops to 36 out of the 200 trials for the 2000 V case. This is a higher likelihood than when only the tissue was a random variable, and approximately the same as when only the tumor was a random variable.

If these simulations were used for treatment planning, it would surely be concluded that 2000 V is insufficient because 90% of the tumor was ablated only 82% of the time. Therefore, a 15% increase in voltage is an insufficient amount for a factor of safety for this setup. However, if we lessen the threshold of ablation to 600 V/cm, then for every trial at least 90% of the tumor or more was ablated.

**Figure 9:**
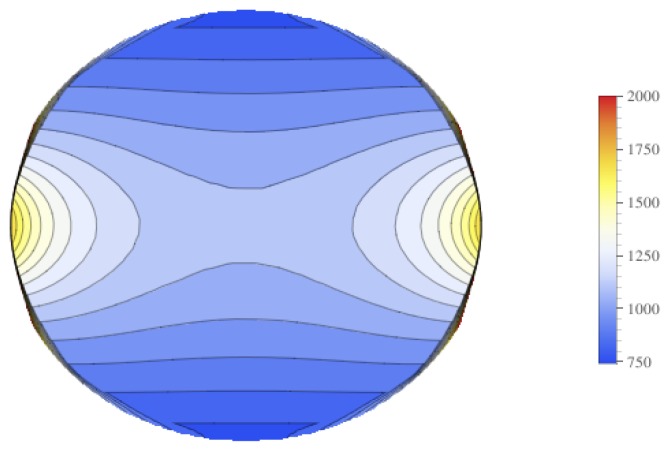
Contour plot of the mean for the electrical field experienced by the Tumor.

**Figure 10:**
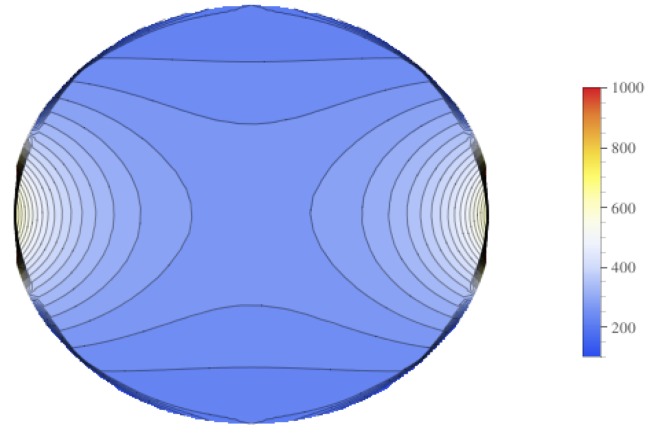
Contour plot of the standard deviation for the electrical field experienced by the Tumor.

**Figure 11:**
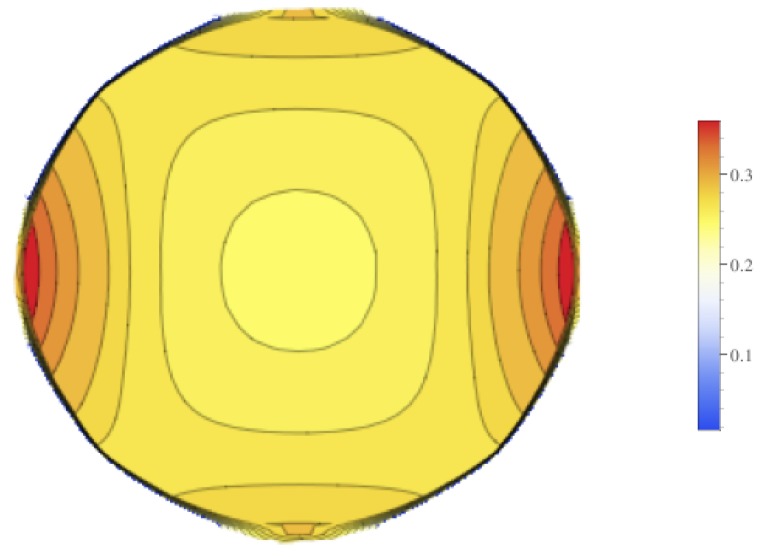
Contour plot of the relative standard deviation for the electrical field experienced by the tumor at 1725 and case 3.

Contour plots of the mean value of the electrical field experienced by the tumor is displayed in Figure 9, and contour plots for the standard deviation of the electrical field is in Figure 10. These figures show that there is higher standard deviation in the electrical field strength near the edges closest to the electrodes, and the least near the poles. This is similar to how the mean of the electrical field is greatest nearest the electrodes and least at the poles. To get a better sense of the percentage of change of the electrical field, the relative standard deviation was plotted in Figure 11. The relative standard deviation is defined to be the standard deviation divided by the mean and is expressed as a percentage. The tumor’s electrical field had a relative standard deviation of approximately 0.2–0.35. The relative standard error was similar for 1800 V and 2000 V.

## 4. Discussion

This paper presented the first attempt at understanding how uncertainty in how an IRE model’s conductivity affects the prediction for the electrical field. This was accomplished by investigating each individual component of parameter uncertainty and then comparing the individual components to the case when all uncertainties are included. Furthermore, this paper contributed by displaying where in the tumor the greatest variability in the electrical field is.

It was found that uncertainty in the tumor conductivity played a smaller role than the uncertainty in the liver tissue when it came to the percentage of the tumor covered by a given electrical field strength. This is potentially good news for treatment planning as tumors have a high variability in conductivity. Conversely, variability in tissue conductivity played a larger role than tumor conductivity in the minimum, maximum and average electrical field strength experienced by the tumor.

Contributions of this paper were:

1. The first UQ for IRE simulations
2. That the relative standard error for the electrical field of the tumor is 0.2–0.3 percent and it’s distribution throughout the tumor
3. That a 15% increase of electrical field resulted in ablating 90% of the tumor only 82% of the time.

Current practice when treatment planning for IRE is to set a margin of safety. It is the author’s belief that the safety margins used in treatment planning should be a reflection of the level of uncertainty in the simulations used for treatment planning. This research aimed to begin to provide a greater understanding of uncertainties so that medical doctors can make more informed decisions when deciding what the safety margins should be.

The goal of this work was not to provide a definitive answer to uncertainty quantification for IRE, but to encourage the application and research of uncertainty quantification in more aspects of treatment planning protocols in irreversible electroporation. IRE is still a relatively new field of study, and as such there is still growing amount of experimental data for conductivity of various tissues. As such there is many opportunities for future research. More specifically, experimental work is needed to better understand the uncertainties of various tissues. More mathematical and numerical work is needed to better understand how these uncertainties propagate into final ablation predictions.

The potential value of this research is to begin to provide expectations of uncertainty in treatment planning and experimental results for IRE. For all three cases, the total area of ablated tissues had a relative standard deviation of ~10%. This amount of uncertainty helps with establishing expectations of accuracy for simulations used in treatment planning of IRE. It is would be reasonable to expect similar standard deviations when comparing experimental IRE procedures on livers to simulations.

This research used uncertainties for conductivity for the liver, but future work could use different organs. Uncertainties in the conductivity is not the same for all tissue, and these different uncertainties in conductivity could be used to develop different factor-of-safety guidelines for different tissues. Future work can also be done to see the effect tumor size has on uncertainty.

In the simulations, it was assumed that the max conductivity for both the tissue and the tumor increased 4.5 times. There is of course uncertainty in the actual value of $\sigma_{max}$ for the tissue and the tumor. Therefore, another area of future work would be to incorporate the uncertainty in how a tissue’s conductivity changes in response to an electrical field.

## Figures and Tables

**Table 1:** Governing equations used to implement the irreversible electroporation (IRE) model.

Equation (1)	∇·(σ∇U)=0
Equation (4)	$\sigma =\sigma_{0}+\frac{\sigma_{max}-\sigma_{0}}{1+exp[\frac{||E||-950}{-200}]}$

**Table 2:** Electrical Field Statistics within the Tumor. Varying $\sigma_{0}$ for liver tissue.

	Average Electrical Field (V/cm)	Min. Electrical Field (V/cm)	Max Electrical Field (V/cm)
$V_{0}=1725$	954±129	688±100	1571±294
$V_{0}=1800$	978±133	706±103	1641±317
$V_{0}=2000$	1042±146	754±109	1844±384

**Table 3:** Ablation zones percentage. Table shows percentage of tumor above a given electrical field strength from varying $\sigma_{0}$ for tissue.

	>1000 V/cm	>800 V/cm	>700 V/cm	>600 V/cm	>500 V/cm
$V_{0}=1725$	39±24	82±21	93±18	97±14	98±11
$V_{0}=1800$	44±25	85±21	94±18	97±14	98±10
$V_{0}=2000$	57±25	90±19	96±16	98±12	99±9

**Table 4:** Ablation sizes for IRE threshold at >700 V/cm.

	Healthy Tissue (mm${}^{2}$)	Tumor & Healthy Tissue (mm${}^{2}$)
$V_{0}=1725$	90.5±6.0	126.3±12.9
$V_{0}=1800$	98.3±6.2	134.5±13.1
$V_{0}=2000$	118.1±6.3	155.0±12.5

**Table 5:** Electrical Field Statistics within the Tumor. Varying $\sigma_{0}$ for the tumor.

	Average Electrical Field (V/cm)	Min. Electrical Field (V/cm)	Max Electrical Field (V/cm)
$V_{0}=1725$	1023±231	737±173	1734±563
$V_{0}=1800$	1051±244	758±179	1814±599
$V_{0}=2000$	1126±278	812±198	2033±694

**Table 6:** Ablation zones percentage. Table shows percentage of tumor above a given electrical field strength from varying $\sigma_{0}$ for the tumor.

	>1000 V/cm	>800 V/cm	>700 V/cm	>600 V/cm	>500 V/cm
$V_{0}=1725$	44±33	82±20	94±9	99±2	100±0
$V_{0}=1800$	48±33	85±17	95±8	99±2	100±0
$V_{0}=2000$	59±31	90±13	98±5	99±1	100±0

**Table 7:** Ablation sizes for IRE threshold at >700 V/cm.

	Healthy Tissue (mm${}^{2}$)	Tumor & Healthy Tissue (mm${}^{2}$)
$V_{0}=1725$	92.2±9.3	128.4±12.8
$V_{0}=1800$	100.0±9.3	136.56±12.4
$V_{0}=2000$	120.5±8.9	158.21±10.8

**Table 8:** Electrical Field Statistics within the Tumor. Varying $\sigma_{0}$ for both liver tissue and the tumor.

	Average Electrical Field (V/cm)	Min. Electrical Field (V/cm)	Max Electrical Field (V/cm)
$V_{0}=1725$	1034±280	744±211	1784±654
$V_{0}=1800$	1064±294	765±218	1868±694
$V_{0}=2000$	1142±332	821±240	2090±805

**Table 9:** Ablation zones percentage. Table shows percentage of tumor above a given electrical field strength from varying $\sigma_{0}$ for both the liver tissue and the tumor.

	>1000 V/cm	>800 V/cm	>700 V/cm	>600 V/cm	>500 V/cm
$V_{0}=1725$	47±37	78±29	89±21	96±14	99±7
$V_{0}=1800$	50±37	80±28	91±20	96±13	99±6
$V_{0}=2000$	59±36	85±24	94±16	98±10	99±4

**Table 10:** Ablation sizes for IRE threshold at >700 V/cm.

	Healthy Tissue (mm${}^{2}$)	Tumor & Healthy Tissue (mm${}^{2}$)
$V_{0}=1725$	92.6±10.9	126.9±19.0
$V_{0}=1800$	100.1±11.3	135.1±19.0
$V_{0}=2000$	120.2±11.7	156.4±17.9

## Abbreviations

The following abbreviations are used in this manuscript:

IRE	Irreversible electroporation
U	Electrical potential
σ	Conductivity
$\sigma_{0}$	Base conductivity
$\sigma_{max}$	max conductivity
E	Electrical field

## References

[1] Yadollahpour A., Rezaee Z. (2014). Electroporation as a New cancer treatment technique: A review on the mechanisms of action. Biomed. Pharmacol. J..

[2] Neumann E., Schaefer-Ridder M., Wang Y., Hofschneider P. (1982). Gene transfer into mouse lyoma cells by electroporation in high electric fields. EMBO J..

[3] Melvik J.E., Pettersen E.O., Gordon P.B., Seglen P.O. (1986). Increase in cis-dichlorodiammineplatinum (II) cytotoxicity upon reversible electropermeabilization of the plasma membrane in cultured human NHIK 3025 cells. Eur. J. Cancer Clin. Oncol..

[4] Mir L.M., Orlowski S., Belehradek J., Paoletti C. (1991). Electrochemotherapy potentiation of antitumour effect of bleomycin by local electric pulses. Eur. J. Cancer Clin. Oncol..

[5] Salford L.G., Persson B., Brun A., Ceberg C., Kongstad P.C., Mir L.M. (1993). A new brain tumor therapy combining bleomycin with in vivo electropermeabilization. Biochem. Biophys. Res. Commun..

[6] Heller R., Jaroszeski M., Leo-Messina J., Perrot R., Van Voorhis N., Reintgen D., Gilbert R. (1995). Treatment of B16 mouse melanoma with the combination of electropermeabilization and chemotherapy. Bioelectrochem. Bioenerg..

[7] Davalos R.V., Mir L., Rubinsky B. (2005). Tissue ablation with irreversible electroporation. Ann. Biomed. Eng..

[8] Thomson K.R., Cheung W., Ellis S.J., Federman D., Kavnoudias H., Loader-Oliver D., Roberts S., Evans P., Ball C., Haydon A. (2011). Investigation of the safety of irreversible electroporation in humans. J. Vasc. Interv. Radiol..

[9] Al-Sakere B., André F., Bernat C., Connault E., Opolon P., Davalos R.V., Rubinsky B., Mir L.M. (2007). Tumor ablation with irreversible electroporation. PLoS ONE.

[10] Sommer C.M., Fritz S., Wachter M.F., Vollherbst D., Stampfl U., Bellemann N., Gockner T., Mokry T., Gnutzmann D., Schmitz A. (2013). Irreversible electroporation of the pig kidney with involvement of the renal pelvis: Technical aspects, clinical outcome, and three-dimensional CT rendering for assessment of the treatment zone. J. Vasc. Interv. Radiol..

[11] Scheltema M.J., Van Den Bos W., de Bruin D.M., Wijkstra H., Laguna M.P., de Reijke T.M., de la Rosette J.J. (2016). Focal vs extended ablation in localized prostate cancer with irreversible electroporation; a multi-center randomized controlled trial. BMC Cancer.

[12] Murray K.S., Ehdaie B., Musser J., Mashni J., Srimathveeravalli G., Durack J.C., Solomon S.B., Coleman J.A. (2016). Pilot Study to Assess Safety and Clinical Outcomes of Irreversible Electroporation for Partial Gland Ablation in Men with Prostate Cancer. J. Urol..

[13] Martin R.C., Kwon D., Chalikonda S., Sellers M., Kotz E., Scoggins C., McMasters K.M., Watkins K. (2015). Treatment of 200 locally advanced (stage III) pancreatic adenocarcinoma patients with irreversible electroporation: Safety and efficacy. Ann. Surg..

[14] Kluger M.D., Epelboym I., Schrope B.A., Mahendraraj K., Hecht E.M., Susman J., Weintraub J.L., Chabot J.A. (2016). Single-institution experience with irreversible electroporation for T4 pancreatic cancer: First 50 patients. Ann. Surg. Oncol..

[15] Barabasch A., Distelmaier M., Heil P., Krämer N.A., Kuhl C.K., Bruners P. (2017). Magnetic Resonance Imaging Findings After Percutaneous Irreversible Electroporation of Liver Metastases: A Systematic Longitudinal Study. Investig. Radiol..

[16] Wendler J., Ganzer R., Hadaschik B., Blana A., Henkel T., Köhrmann K., Machtens S., Roosen A., Salomon G., Sentker L. (2016). Why we should not routinely apply irreversible electroporation as an alternative curative treatment modality for localized prostate cancer at this stage. World J. Urol..

[17] Vasilkoski Z., Esser A.T., Gowrishankar T., Weaver J.C. (2006). Membrane electroporation: The absolute rate equation and nanosecond time scale pore creation. Phys. Rev. E.

[18] Golberg A., Rubinsky B. (2010). A statistical model for multidimensional irreversible electroporation cell death in tissue. Biomed. Eng. Online.

[19] Weaver J.C. (2003). Electroporation of biological membranes from multicellular to nano scales. IEEE Trans. Dielectr. Electr. Insul..

[20] Ivorra A., Al-Sakere B., Rubinsky B., Mir L.M. (2009). In vivo electrical conductivity measurements during and after tumor electroporation: Conductivity changes reflect the treatment outcome. Phys. Med. Biol..

[21] Corovic S., Lackovic I., Sustaric P., Sustar T., Rodic T., Miklavcic D. (2013). Modeling of electric field distribution in tissues during electroporation. Biomed. Eng. Online.

[22] Neal R.E., Garcia P.A., Kavnoudias H., Rosenfeldt F., Mclean C.A., Earl V., Bergman J., Davalos R.V., Thomson K.R. Simulation of In Vivo Irreversible Electroporation Renal Ablations. Proceedings of the 6th European Conference of the International Federation for Medical and Biological Engineering.

[23] Banús Cobo J. 3D assessment of irreversible electroporation treatments in vegetal models. Proceedings of the 1st World Congress on Electroporation and Pulsed Electric Fields in Biology, Medicine and Food & Environmental Technologies.

[24] Qasrawi R., Silve L., Burdío F., Abdeen Z., Ivorra A. (2017). Anatomically Realistic Simulations of Liver Ablation by Irreversible Electroporation: Impact of Blood Vessels on Ablation Volumes and Undertreatment. Technol. Cancer Res. Treat..

[25] Ivorra A., Mir L., Rubinsky B. Electric field redistribution due to conductivity changes during tissue electroporation: Experiments with a simple vegetal model. Proceedings of the World Congress on Medical Physics and Biomedical Engineering.

[26] Jourabchi N., Beroukhim K., Tafti B.A., Kee S.T., Lee E.W. (2014). Irreversible electroporation (NanoKnife) in cancer treatment. Gastrointest. Interv..

[27] Edd J.F., Davalos R.V. (2008). Mathematical Modeling of Irreversible Electroporation for Treatment Planning. Technol. Cancer Res. Treat..

[28] Haemmerich D., Schutt D.J., Wright A.S., Webster J.G., Mahvi D.M. (2009). Electrical conductivity measurement of excised human metastatic liver tumours before and after thermal ablation. Physiol. Meas..

[29] Miklavčič D., Šemrov D., Mekid H., Mir L.M. (2000). A validated model of in vivo electric field distribution in tissues for electrochemotherapy and for DNA electrotransfer for gene therapy. Biochim. Biophys. Acta.

[30] Sano M.B., Arena C.B., DeWitt M.R., Saur D., Davalos R.V. (2014). In-vitro bipolar nano-and microsecond electro-pulse bursts for irreversible electroporation therapies. Bioelectrochemistry.

[31] Daniels C., Rubinsky B. (2009). Electrical field and temperature model of nonthermal irreversible electroporation in heterogeneous tissues. J. Biomech. Eng..

[32] Xie F., Zemlin C.W. (2016). Effect of Twisted Fiber Anisotropy in Cardiac Tissue on Ablation with Pulsed Electric Fields. PLoS ONE.

[33] Zhang Y., Guo Y., Ragin A.B., Lewandowski R.J., Yang G.Y., Nijm G.M., Sahakian A.V., Omary R.A., Larson A.C. (2010). MR Imaging to Assess Immediate Response to Irreversible Electroporation for Targeted Ablation of Liver Tissues: Preclinical Feasibility Studies in a Rodent Model 1. Radiology.

[34] Mandel Y., Rubinsky B. (2012). Treatment of Uveal Melanoma by Nonthermal Irreversible Electroporation: Electrical and Bioheat Finite Element Model of the Human Eye. J. Heat Transf..

[35] Wimmer T., Srimathveeravalli G., Gutta N., Ezell P.C., Monette S., Maybody M., Erinjery J.P., Durack J.C., Coleman J.A., Solomon S.B. (2015). Planning irreversible electroporation in the porcine kidney: Are numerical simulations reliable for predicting empiric ablation outcomes?. Cardiovasc. Interv. Radiol..

